# ICG-guided bowel preservation during emergency TAPP repair for incarcerated Spigelian hernia: a case report

**DOI:** 10.1093/jscr/rjag701

**Published:** 2026-08-12

**Authors:** Hiroto Sakurai, Shingo Tsujinaka, Kenichiro Yambe, Kazuhiro Takami, Noriko Kondo, Kei Nakagawa, Kuniharu Yamamoto, Toru Nakano, Chikashi Shibata, Yu Katayose

**Affiliations:** Division of Hepato-Biliary and Pancreatic Surgery, Department of Surgery, Tohoku Medical and Pharmaceutical University, Sendai, Miyagi, Japan; Division of Gastroenterologic Surgery, Department of Surgery, Tohoku Medical and Pharmaceutical University, Sendai, Miyagi, Japan; Division of Hepato-Biliary and Pancreatic Surgery, Department of Surgery, Tohoku Medical and Pharmaceutical University, Sendai, Miyagi, Japan; Division of Hepato-Biliary and Pancreatic Surgery, Department of Surgery, Tohoku Medical and Pharmaceutical University, Sendai, Miyagi, Japan; Division of Hepato-Biliary and Pancreatic Surgery, Department of Surgery, Tohoku Medical and Pharmaceutical University, Sendai, Miyagi, Japan; Division of Hepato-Biliary and Pancreatic Surgery, Department of Surgery, Tohoku Medical and Pharmaceutical University, Sendai, Miyagi, Japan; Division of Hepato-Biliary and Pancreatic Surgery, Department of Surgery, Tohoku Medical and Pharmaceutical University, Sendai, Miyagi, Japan; Division of Gastroenterologic Surgery, Department of Surgery, Tohoku Medical and Pharmaceutical University, Sendai, Miyagi, Japan; Division of Gastroenterologic Surgery, Department of Surgery, Tohoku Medical and Pharmaceutical University, Sendai, Miyagi, Japan; Division of Hepato-Biliary and Pancreatic Surgery, Department of Surgery, Tohoku Medical and Pharmaceutical University, Sendai, Miyagi, Japan

**Keywords:** Spigelian hernia, incarcerated hernia, indocyanine green fluorescence, transabdominal preperitoneal approach, bowel viability

## Abstract

Spigelian hernia (SpH) is a rare abdominal wall hernia associated with a significant risk of incarceration. We report the case of an incarcerated SpH that was successfully managed with emergency laparoscopic surgery. An 81-year-old female presented with acute abdominal pain. Contrast-enhanced computed tomography revealed small bowel incarceration through a defect lateral to the left rectus abdominis muscle, with preserved blood flow. After unsuccessful manual reduction, emergency laparoscopic surgery was performed. The incarcerated bowel was reduced using a hydrostatic reduction technique with gentle traction. Serial indocyanine green fluorescence angiography demonstrated improving bowel perfusion, and bowel resection was avoided. The hernia was repaired via the transabdominal preperitoneal (TAPP) approach with defect closure. The postoperative course was uneventful, and no recurrence was observed during 3.5 years of follow-up. Emergency TAPP repair combined with serial indocyanine green-guided perfusion assessment may be a useful option for carefully selected cases of incarcerated SpH.

## Introduction

A Spigelian hernia (SpH) is a rare type of ventral hernia, accounting for ~2% of all abdominal wall hernias [1, 2]. Due to the typically small size of the hernia defect (<2 cm) and its confinement by rigid aponeurosis, SpH carries a relatively high risk of incarceration, reported in 17%–24% of cases [1]. Although the laparoscopic repair of SpH, including the transabdominal preperitoneal (TAPP) approach, has been increasingly documented [1–4], evidence regarding the emergency repair of incarcerated SpH remains limited. Furthermore, reports detailing the use of intraoperative indocyanine green (ICG) fluorescence imaging for assessing bowel viability in this context are extremely scarce. Here, we present a case of incarcerated SpH that was successfully managed via emergency TAPP repair with ICG-guided bowel preservation and no postoperative complications or recurrence during follow-up.

## Case report

**Figure 1:**
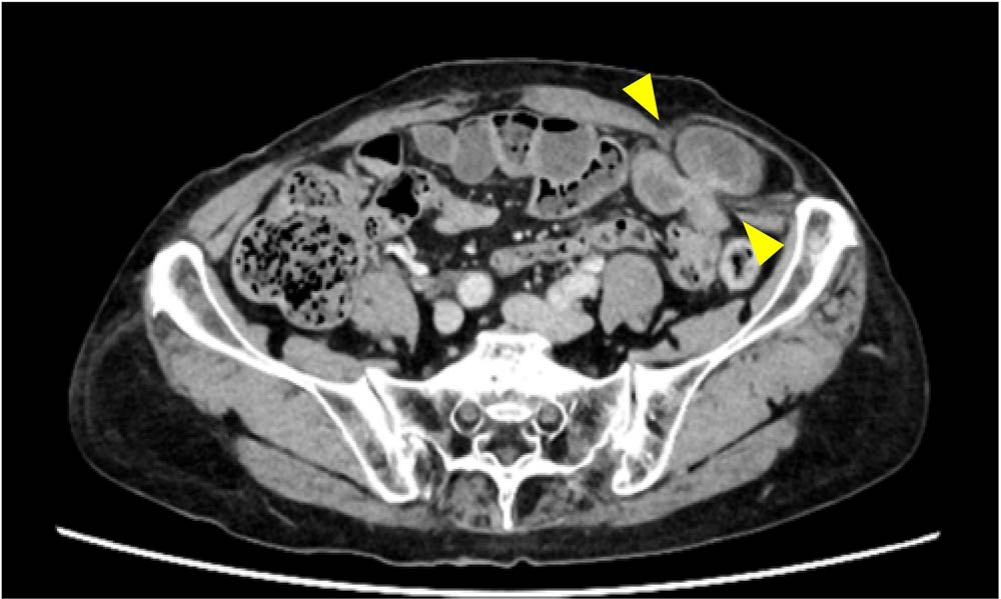
Preoperative contrast-enhanced CT revealed small bowel prolapse through a defect lateral to the left rectus abdominis muscle, with preserved enhancement suggesting maintained perfusion (arrowheads).

An 81-year-old female presented with acute abdominal pain. Past medical history included diabetes mellitus, hyperlipidemia, cutaneous arteritis, and dermatomyositis requiring long-term steroid treatment with temporary dose escalation for disease control. She had previously undergone surgery for appendicitis and lumbar spinal canal stenosis. Physical examination revealed a tender, palpable mass measuring 5 cm in the left lower quadrant. Blood test results were within normal limits. Contrast-enhanced abdominal computed tomography (CT) revealed small bowel prolapse through the left abdominal wall. The hernia orifice was located lateral to the rectus abdominis muscle, within the Spigelian belt [1, 5], raising suspicion of an incarcerated SpH. Blood flow to the incarcerated small bowel appeared preserved (Fig. 1).

**Figure 2:**
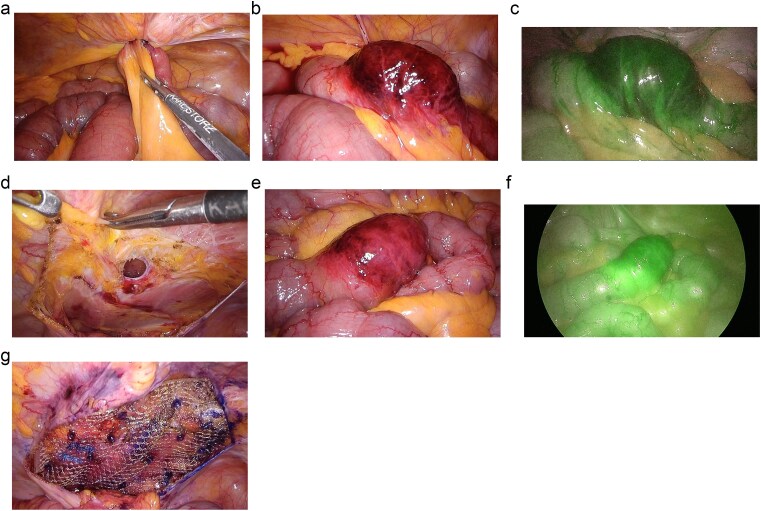
(a) Incarcerated Richter-type hernia. Laparoscopic view of the incarcerated small bowel within the defect, consistent with a Richter-type hernia, which could not be reduced manually. (b) Assessment of bowel perfusion under white-light observation. Following reduction, the bowel appeared congested; however, the presence of peristalsis suggested reperfusion. (c) Assessment of bowel perfusion under indocyanine green fluorescence imaging. Indocyanine green fluorescence angiography showed patchy perfusion of the affected bowel segment, suggesting possible reperfusion and requiring serial assessment. (d) Exposure of the hernia defect. Dissection of the preperitoneal space with retraction of preperitoneal fat, clearly exposing the hernia defect. (e) Second assessment of bowel perfusion under white-light observation. Repeat assessment demonstrated improved serosal color of the bowel, suggesting recovery of perfusion. (f) Second assessment of bowel perfusion by indocyanine green fluorescence imaging. Repeat indocyanine green imaging demonstrated improved and more uniform vascularization of the previously affected segment. (g) Mesh placement and fixation. The mesh was positioned within the preperitoneal space and secured with absorbable tacks.

Due to unsuccessful manual reduction, emergency laparoscopic surgery was indicated. Under general anesthesia, the patient was positioned supine, and the procedure was performed using three ports. Laparoscopic exploration revealed that the hernia defect was situated between the lateral edge of the left rectus abdominis muscle and the semilunar line. The small bowel was incarcerated in the defect, suggestive of a Richter-type hernia, and could not be manually reduced (Fig. 2a). A hydrostatic reduction technique was then attempted using an 8-Fr Nelaton catheter and 20 ml of saline. The injection pressure was not measured; however, this approach proved inadequate, and the bowel was ultimately reduced with gentle traction. Although the reduced bowel appeared congested, immediate peristalsis was observed under white-light inspection (Fig. 2b). After intravenous administration of ICG (5 mg), fluorescence angiography using the VISERA ELITE II system (Olympus, Tokyo, Japan) showed a patchy perfusion pattern [6], suggesting possible bowel reperfusion but not providing definitive evidence of bowel viability (Fig. 2c). Because peristalsis had recovered and no segment with complete absence of fluorescence was observed, immediate bowel resection was deferred, and serial assessment was performed.

The hernia was repaired using the TAPP approach. Following complete inversion of the hernia sac, the peritoneum was incised above the defect, and the preperitoneal space was dissected. The preperitoneal fat protruding into the defect was retracted, clearly exposing the hernia defect (Fig. 2d). The defect measured ~1 cm in diameter. Dissection was continued to achieve a 4- to 5-cm overlap. The hernia defect was closed with a continuous 3-0 barbed suture (Quill™, Corza Medical, Boulder, CO, USA). A 3D Max™ Light Mesh (large size: 10.3 × 15.7 cm) (Becton, Dickinson and Company, Franklin Lakes, NJ, USA) was selected and trimmed to cover the dissected area. A second bowel assessment with ICG fluorescence angiography (ICG, 5 mg) revealed improved and more uniform fluorescence, together with improved gross appearance (Figs. 2e and f). Based on these serial findings, the bowel was judged viable, and bowel resection was avoided. The mesh was positioned and secured with absorbable tacks (Fig. 2g). The peritoneum was closed with a continuous 3-0 barbed suture (V-Loc™, Medtronic, Minneapolis, MN, USA). The operative time was 113 minutes. The intraoperative findings and key steps of the surgical procedure are presented in Supplementary Video 1.

**Figure 3:**
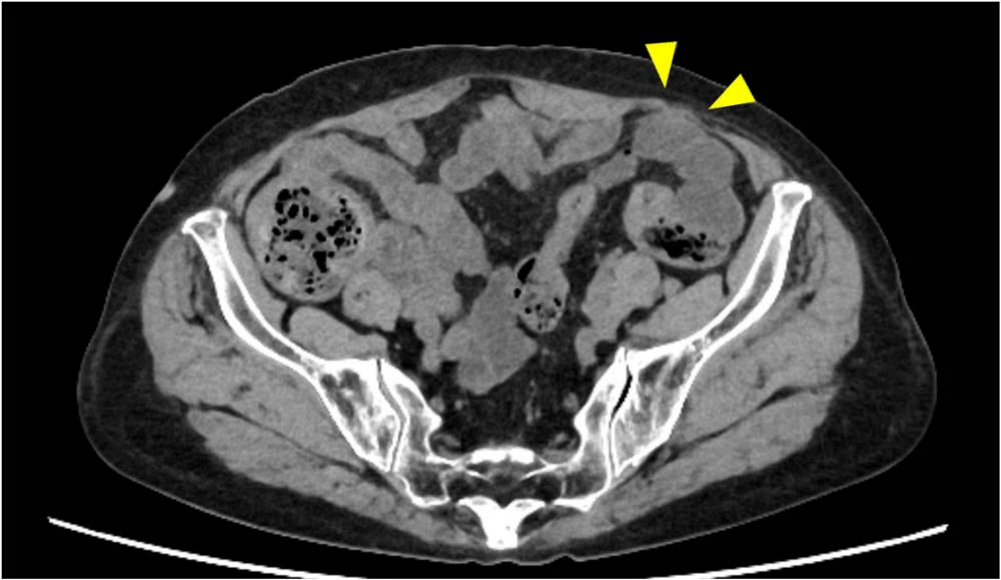
CT at the 6-month follow-up showed no evidence of hernia recurrence at the repair site (arrowheads).

The postoperative course was uneventful, and the patient was discharged on postoperative Day 3. A CT conducted 6 months postoperatively showed no evidence of hernia recurrence (Fig. 3). At 3.5 years postoperatively, the patient remains free of abdominal symptoms and hernia recurrence.

## Discussion

Emergency surgery for incarcerated SpH is infrequently encountered, making it practical to utilize surgical techniques that are familiar and routinely employed in other laparoscopic settings. In the present case, the combination of the hydrostatic reduction technique, ICG fluorescence angiography, and TAPP repair facilitated safe bowel reduction, assessment of bowel viability, and standardized hernia repair.

The hydrostatic reduction technique was used as an adjunctive maneuver to minimize direct traction on the incarcerated bowel [7]. In this case, 20 ml of saline was injected through an 8-Fr Nelaton catheter; however, the injection pressure was not measured, and hydrostatic reduction alone was insufficient. Although this technique may facilitate atraumatic reduction in selected cases, standardized pressure or volume parameters have not been established [7]. Therefore, it should be performed cautiously and should not be continued when marked resistance, bowel fragility, perforation, or irreversible ischemia is suspected. After the reduction of a Richter-type hernia, evaluating the viability of the incarcerated bowel can often be challenging through white-light observation alone. ICG fluorescence angiography has increasingly been employed for intraoperative assessment of bowel ischemia in strangulated bowel obstruction, non-occlusive mesenteric ischemia, and incarcerated hernias [6, 8–10]. Ryu *et al*. [11] further suggested that ICG fluorescence angiography may be useful for evaluating intestinal blood flow during laparoscopic surgery for strangulated bowel obstruction and may substitute for direct palpation in selected laparoscopic settings. Moreover, a recent case report also described ICG-guided bowel preservation in Richter-type SpH treated by a combined laparoscopic and open approach [12]. In the present case, the initial patchy fluorescence was not interpreted as definitive evidence of bowel viability, although a previous report suggested that a patchy pattern could correspond to pathologically viable bowel in a resected specimen [6]. Rather, the absence of complete non-fluorescence, recovery of peristalsis, and subsequent improvement in fluorescence and gross appearance supported bowel preservation. Therefore, ICG imaging may serve as an adjunctive tool in determining the necessity for bowel resection when bowel viability is uncertain under white-light observation.

TAPP was selected because there was no evidence of bowel necrosis, perforation, or gross contamination, and bowel preservation appeared possible after reduction. This approach enabled direct inspection of the incarcerated bowel and preperitoneal mesh placement with peritoneal closure, thereby avoiding intraperitoneal mesh exposure. Compared with the intraperitoneal onlay mesh technique (IPOM), TAPP may offer the advantages of preperitoneal mesh placement and avoidance of direct intraperitoneal mesh contact in selected small abdominal wall hernias [13]. However, open repair or laparotomy should be considered when bowel necrosis, perforation, severe contamination, hemodynamic instability, or inadequate laparoscopic working space is present [14]. Therefore, emergency TAPP should only be considered in selected cases after careful assessment of bowel viability and by surgeons with adequate laparoscopic expertise.

Long-term steroid therapy may raise concerns regarding impaired wound healing and susceptibility to infection. In abdominal wall hernia repair, steroid use has been identified as a significant risk factor for surgical mesh infection [15]. In the present case, however, bowel resection was avoided, and there was no perforation or gross contamination. Therefore, preperitoneal mesh placement with peritoneal closure was considered acceptable. Nevertheless, mesh repair in immunosuppressed or steroid-dependent patients should be selected cautiously according to the degree of contamination, need for bowel resection, and overall patient risk.

This report has several limitations. First, it constitutes a single case report, and the strategy applied herein may not be generalizable. Second, the effectiveness of emergency TAPP and ICG assessment relies on the surgeon’s expertise, the availability of equipment, and institutional readiness. Third, the ICG assessment in this case was qualitative rather than quantitative; standardized thresholds have not been established, and fluorescence patterns may depend on the imaging device used intraoperatively. Therefore, these findings should be interpreted as complementary to conventional gross evaluation. Further accumulation of cases is necessary to clarify the indications, reproducibility, and applicability of the presented strategy for incarcerated SpH.

In conclusion, emergency TAPP repair with ICG-guided assessment of bowel perfusion may represent an option for carefully selected cases of incarcerated SpH, as it enabled bowel preservation with no recurrence during 3.5 years of follow-up in this case. However, given the nature of a single case report, broader applicability should be interpreted with caution.

## Supplementary Material

Supplementary_material_rjag701
