# Supplementary material for: ICG-guided bowel preservation during emergency TAPP repair for incarcerated Spigelian hernia: a case report
Source: J Surg Case Rep. 2026 Aug 12;2026(8):rjag701. doi: 10.1093/jscr/rjag701 (PMC13464707; doi:10.1093/jscr/rjag701)
Supplement: Supplementary file 1 [file supplementary_material_rjag701.zip › Supplementary Video 1_narration_Incarcerated SpH.docx]

**Video narration**

- Laparoscopic exploration showed that the hernia defect was located between the lateral edge of the left rectus abdominis muscle and the semilunar line. The procedure was performed using three ports.
- The small bowel was incarcerated in the hernia defect. A water pressure method was attempted using an 8-French Nelaton catheter and 20 milliliters of saline. The bowel was then reduced with gentle traction.
- This was a Richter-type hernia. Although congestion remained, peristalsis was immediately observed, suggesting restoration of blood flow.
- The hernia sac was completely inverted.
- ICG fluorescence angiography showed a patchy perfusion pattern. Based on this finding, bowel resection was not performed.
- Hernia repair was performed using the TAPP approach. The peritoneum was incised above the defect, and the preperitoneal space was dissected.
- The preperitoneal fat entering to the hernia defect was pulled out, clearly exposing the hernia defect. Dissection continued to obtain 4- to 5-centimeter overlap.
- The hernia defect was closed with a continuous 3-0 barbed suture.
- A 3D Max Light mesh was selected and trimmed to cover the dissected area.
- A second ICG fluorescence angiography demonstrated improved vascularization, confirming restoration of bowel perfusion.
- The mesh was placed and fixed with absorbable tacks.
- The peritoneum was then closed with a continuous 3-0 barbed suture.
